# Effectiveness of a healthcare-based mobile intervention on sedentary patterns, physical activity, mental well-being and clinical and productivity outcomes in office employees with type 2 diabetes: study protocol for a randomized controlled trial

**DOI:** 10.1186/s12889-022-13676-x

**Published:** 2022-06-29

**Authors:** Francesc Alòs, Mª. Àngels Colomer, Carlos Martin-Cantera, Montserrat Solís-Muñoz, Judit Bort-Roig, I. Saigi, E. Chirveches-Pérez, Mercè Solà-Gonfaus, Josep Maria Molina-Aragonés, Anna Puig-Ribera

**Affiliations:** 1 Primary Healthcare Centre Passeig de Sant Joan, Catalan Health Institute, 08010 Barcelona, Spain; 2 Department of Mathematics, ETSEA, University of Lleida, Lleida, Spain; 3 Barcelona Research Support Unit, Primary Care Research Institute IDIAP Jordi Gol, Barcelona, Spain; 4 Health Care Research Unit, Puerta de Hierro Majadahonda University Hospital. Nursing and Health Care Research Group, Puerta de Hierro-Segovia de Arana, Health Research Institute, Madrid, Spain; 5 Sport and Physical Activity Research Group, Centre for Health and Social Care Research, University of Vic-Central University of Catalonia, Vic, Spain; 6 Endocrinology and Nutrition Department, Vic University Hospital, Barcelona, Spain; 7 Research Group on Methodology, Methods, Models and Outcomes of Health and Social Sciences, Centre for Health and Social Care Research, University of Vic-Central University of Catalonia, Barcelona, Spain; 8 Primary Healthcare Centre Les Planes, Catalan Health Institute, Barcelona, Spain; 9 Health and Prevention Department, Catalan Health Institute, Barcelona, Spain

**Keywords:** Mobile applications, Smartphone, Sedentary behaviour, Physical activity, Office employees, Primary healthcare, Diabetes mellitus type 2, Workplace

## Abstract

**Background:**

Prolonged sedentary time is associated with an increased incidence of chronic disease including type 2 diabetes mellitus (DM2). Given that occupational sedentary time contributes significantly to the total amount of daily sedentariness, incorporating programmes to reduce occupational sedentary time in patients with chronic disease would allow for physical, mental and productivity benefits. The aim of this study is to evaluate the short-, medium- and long-term effectiveness of a mHealth programme for sitting less and moving more at work on habitual and occupational sedentary behaviour and physical activity in office staff with DM2. Secondary aims. To evaluate the effectiveness on glycaemic control and lipid profile at 6- and 12-month follow-up; anthropometric profile, blood pressure, mental well-being and work-related post-intervention outcomes at 3, 6 and 12 months.

**Methods:**

Multicentre randomized controlled trial. A sample size of 220 patients will be randomly allocated into a control (*n* = 110) or intervention group (n = 110), with post-intervention follow-ups at 6 and 12 months. Health professionals from Spanish Primary Health Care units will randomly invite patients (18–65 years of age) diagnosed with DM2, who have sedentary office desk-based jobs. The control group will receive usual healthcare and information on the health benefits of sitting less and moving more. The intervention group will receive, through a smartphone app and website, strategies and real-time feedback for 13 weeks to change occupational sedentary behaviour. Variables: (1) Subjective and objective habitual and occupational sedentary behaviour and physical activity (Workforce Sitting Questionnaire, Brief Physical Activity Assessment Tool, activPAL3TM); 2) Glucose, HbA1c; 3) Weight, height, waist circumference; 4) Total, HDL and LDL cholesterol, triglycerides; (5) Systolic, diastolic blood pressure; (6) Mental well-being (Warwick-Edinburgh Mental Well-being); (7) Presenteeism (Work Limitations Questionnaire); (8) Impact of work on employees´ health, sickness absence (6th European Working Conditions Survey); (9) Job-related mental strain (Job Content Questionnaire). Differences between groups pre- and post- intervention on the average value of the variables will be analysed.

**Discussion:**

If the mHealth intervention is effective in reducing sedentary time and increasing physical activity in office employees with DM2, health professionals would have a low-cost tool for the control of patients with chronic disease.

**Trial Registration:**

ClinicalTrials.gov NCT04092738. Registered September 17, 2019.

**Supplementary Information:**

The online version contains supplementary material available at 10.1186/s12889-022-13676-x.

## Background

Sedentary behaviour and physical inactivity are associated with an increased risk of chronic disease [1–3] and all-cause mortality [4, 5]. Prolonged sitting is associated with weight gain and obesity [6], metabolic syndrome [7, 8], cardiovascular disease [5, 9], a higher incidence of DM2 [6, 7, 10] and some types of cancer [11–13]. Excessive sitting is also associated with mental health problems such as lower mental well-being, anxiety and increased risk of depression [14–16]. In modern societies, these unhealthy behaviours contribute to chronic diseases, which progress slowly, appear increasingly early and lead to a loss in quality of life, social labour and health costs [17, 18]. Sedentary behaviour is defined as any activity with a caloric expenditure ≤1.5 Metabolic Equivalent Tasks while remaining in a sitting or reclining posture [19]. Physical inactivity, in turn, is defined as an insufficient physical activity level to meet physical activity recommendations [20]. Sedentary behaviour is a health-related risk factor regardless of physical inactivity [21], which has become very prevalent worldwide due to changes in the physical, social and economic environment [22, 23].

Worldwide, Ding et al. [24] estimated that physical inactivity cost health systems $53.8 billion in 2013. In addition, deaths attributable to physical inactivity cost a further $13.7 billion in lost productivity and resulted in 13.4 million disability-adjusted life years. It was also estimated that sedentary behaviour cost the National Health Service of the United Kingdom (2016–2017) £0.8 billion, which included expenditures on cardiovascular diseases (£424 million), DM2 (£281 million) and colon, lung and endometrial cancers (£56 million) [25]. In spite of that, longitudinal data (2007–2016) on adults from the United States [26] did not observe a significant increase in the adherence rate (63.2 and 65.2% respectively) to the physical activity recommendations (e.g. at least 150 minutes of moderate-intensity or 75 minutes of vigorous-intensity aerobic physical activity per week or some equivalent combination) [20] but a significant increase in sedentary behaviour. The COVID-19 pandemic has promoted physical inactivity and sedentary behaviour [27–29], which had been defined as another pandemic [27, 30] and a major public health problem that must be addressed jointly by healthcare professionals [31].

Although there is no international consensus with regards to healthy sedentary behaviour thresholds for adults, a meta-analysis suggested that adults’ sedentary time should be limited to 7-8 h/day [4]. Other public health guidelines also recommend breaking up sedentary behaviour every 30 minutes [32]. And the World Health Organization (WHO, 2020) [20] suggests that adults should limit the amount of time they spend sedentary.

### Occupational sedentary behaviour, physical inactivity and health

Evidence on the specific health effects of physical activity and sedentary behaviour in the occupational domain are less conclusive [33, 34]. Many adults spend half or more of their day at work, and the hours they spend sitting there make up half of their total sedentary time [35]. Moreover, the COVID-19 pandemic has promoted teleworking and therefore an increase in occupational sedentary behaviour, such as office work at home [36, 37]. Consequently, it is estimated that sedentary time at work would be responsible for 50% of the negative health effects attributable to sedentary behaviour [38].

Additionally, sedentary work time is associated with indicators of productivity and well-being [39], suggesting that “sitting less and moving more” at work could effectively reduce an array of markers of lost productivity [40]. This is especially relevant in people with prevalent chronic disease, who show higher losses of work productivity [41]. “Sitting less and moving more” could also be effective to cope with the increasing levels of work-related stress [42], another current major challenge for people with chronic disease [42, 43]. For all the above, reducing sedentary behaviour at the workplace is a priority area in which to intervene.

Reducing occupational sedentary time by promoting incidental physical activity (such as climbing stairs or walking for short periods) is a key prevention strategy that could help people gradually increase their physical activity levels towards those recommended for optimum health [44, 45]. While numerous health and financial benefits for both employees and employers could be achieved [44, 45], the evidence on effective, long-term intervention strategies in the workplace to reduce sedentary behaviour is limited [46, 47].

### Mobile Health (mHealth) interventions to reduce occupational sedentary behaviour and physical inactivity

Mobile health (mHealth) has been defined as medical and public health practice supported by mobile devices such as mobile phones, patient monitoring devices, and personal digital assistants [48]. mHealth programmes could help change time spent on sedentary behaviour [49–51]. mHealth interventions at work are feasible, acceptable and effective tools for promoting physical activity [49] and reducing sedentary behaviour, even outside working hours [52]. However, studies evaluating the impact of these long-term and specific mHealth programmes on occupational sedentary behaviour are low [49].

Furthermore, the use and efficacy of mHealth programmes mostly focus on healthy adults, without taking into account the impact they might have on specific population groups [53], such as workers with prevalent chronic diseases like obesity or DM2. There is a need for a better understanding of how to integrate mHealth programmes in the self-management of patients with chronic diseases, specifically in the self-management of DM2, and in health care in general [54]. Self-management is fundamental for the well-being of people with DM2 [55, 56] who are mostly treated and managed in primary health care. mHealth interventions as a means of self-management and health promotion offer a promising solution in primary health care to cope with the increasing demand for treatment and control of DM2 [55].

### Healthcare-based mHealth interventions to reduce occupational sedentary behaviour and physical inactivity in people with chronic diseases

Primary health care plays a key role in promoting physical activity and reducing sedentary behaviour for chronic disease. Physical activity is a cost-effective drug that is universally prescribed as first-line treatment, especially to patients with chronic diseases like DM2 [57]. Patients’ physical activity levels and sedentary behaviour reductions can be achieved by using new strategies in clinical practice such as technology including wearables and mHealth programmes. These can be useful for improving treatment adherence and enabling the evaluation and registration of lifestyles in the medical records [57].

### Objectives

In this context, the main objective of this protocol for a randomized controlled trial is to assess the effectiveness in the short-medium and long term of a mHealth programme to “sit less and move more” at work on reducing sedentary behaviour and increasing habitual and work physical activity in office staff with DM2. The secondary objectives are to evaluate the effectiveness of the programme on i) clinical variables: glycaemic control and lipid profile; ii) anthropometric and blood pressure variables; iii) improvement in mental well-being, presenteeism, absenteeism and work-related stress.

This is important given the scarcity of studies that evaluate the effectiveness of mHealth programmes in reducing sedentary behaviour as a therapeutic approach for people with chronic pathology, specifically with DM2, which also evaluate the global health impact of the mHealth intervention on clinical parameters, mental well-being and productivity [46]. This study will make it possible to clarify whether people diagnosed with one of the most prevalent chronic diseases, DM2, are likely to benefit from the implementation of mHealth interventions by healthcare professionals in a wide range of health variables and through the use of objective measures of habitual and occupational sedentary behaviour [49].

### Trial design

This is a prospective, multicentre, two-arm randomized controlled trial with an intervention and control group (i.e. usual care) that will evaluate the effectiveness of an intervention based on a 13-week mHealth programme that aims to replace sedentary work tasks with active ones in office workers with DM2.

## Methods

This study protocol has been developed based on the Standard Protocol Items: Recommendation for Interventional Trials (SPIRIT) guidelines [58].

### Study setting

Data will be collected from primary health care centres in the metropolitan area of Barcelona. This densely populated urban area has a high prevalence of patients with DM2 - in Spain, 7.8% of the adult population have DM2 from which 4.17% belong to the age range of 18–65 years old (5.35% males, 3.28% females) [59] – which ensures easy access to people with this medical condition.

### Recruitment

First, project information will be disseminated to the directors of the primary health care centres in the aforementioned metropolitan area through the Foundation University Institute for Research in Primary Health Care Jordi Gol i Gurina. A meeting with the medical staff of each general practice that shows an interest in volunteering for the project will be organized to recruit physicians and nurses willing to participate in the project. Physicians and nurses that volunteer will sign a written informed consent agreeing to recruit and monitor 5–6 patients for each medical staff unit. Each unit will include one physician and one nurse.

General practices recruitment will be ongoing until enough medical units are reached to achieve the required sample size. In general practices where medical units will participate, a member of the research team will run a two-hour training course with the medical staff that will include: (i) Benefits of physical activity and reduction of sedentary behaviour in DM2; (ii) Information and objectives of the study; (iii) Schedule of the visits and tasks in each follow-up visit. Also, a certified medical professional will provide medical units with a full list of adult patients (18–65 years of age) within their own patients’ portfolio that are diagnosed with DM2 and could potentially participate in the study. Medical units will be advised to ring patients during the first week after the training session, following the list of names order and asking for the inclusion criteria. A specific interview guide for physicians and nurses will be developed to standardize recruitment procedures and identify patients that meet the inclusion criteria.

**Fig. 1:**
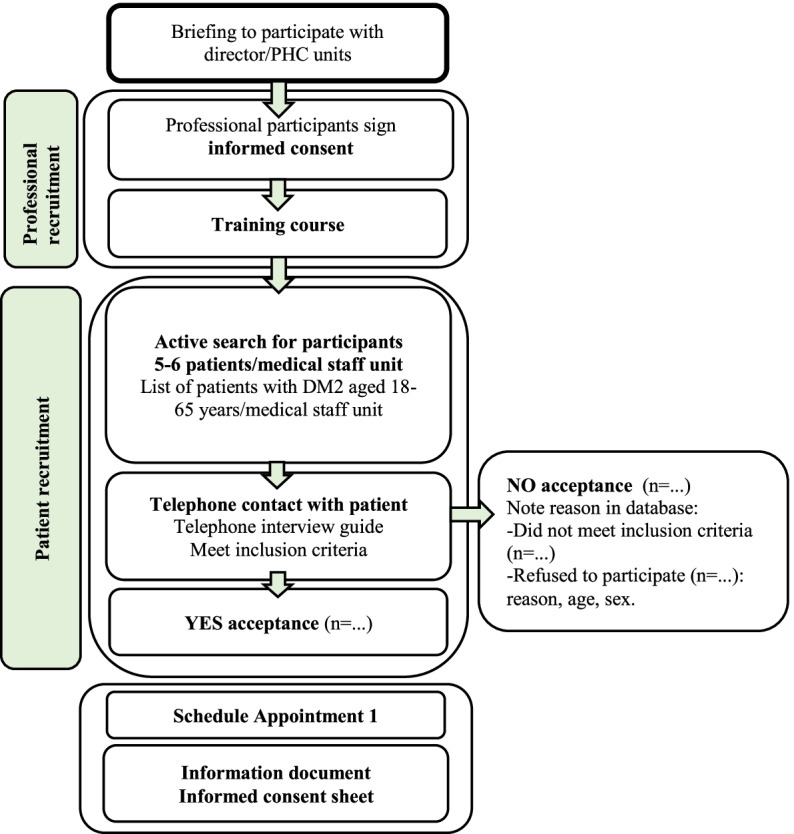
Recruitment procedure for the Walk@Work-App study protocol of a randomized controlled trial

Patients that meet the inclusion criteria will be invited to participate. If the response is NO, reasons for not volunteering will be recorded in an on-line form (Survey Monkey Enterprise). If the response is YES, more detailed information about the study will be provided and a first appointment with the nurse will be organized in the general practice. In appointment 1, patients will be provided with written detailed information about the study, and if they volunteer they will sign a written informed consent to participate. This process will be repeated until each unit recruits the 5–6 patients agreed (Fig. 1).

### Eligibility criteria

Participating patients must be between 18 and 65 years old (working age) and have a mobile phone (smartphone). They must also be diagnosed with DM2 in accordance with international criteria [60], be office workers with a minimum of 55% of their daily working hours performing sedentary tasks according to the Occupational Sitting and Physical Activity Questionnaire (OSPAQ) [61] and have a work contract of at least 18.5 hours.

Furthermore, participants must not (i) have a diagnosis of musculoskeletal, cardiovascular, pulmonary or orthopaedic problems or any other physical condition that prevents them from being physically active; (ii) participate simultaneously in another study or programme of sedentary behaviour, physical activity, nutrition or weight control; (iii) be pregnant; or (iv) have a history of psychiatric problems or substance abuse that could interfere with adherence to the study protocol.

### Allocation

After obtaining the informed consent of the patients to participate in the trial, they will be randomly assigned to the control and intervention group by the research team. The research team will use an Excel file to create a random sequence of numbers allocating patients into two blocks: the control and the intervention group. The sequence of numbers will be the result of a simple randomization process. The research team will randomly provide each medical unit with a series of six numbers to allocate to each patient. After finishing recruiting, the medical unit will allocate one number to each patient and will send this information to the research team.

### Blinding

**Fig. 2:**
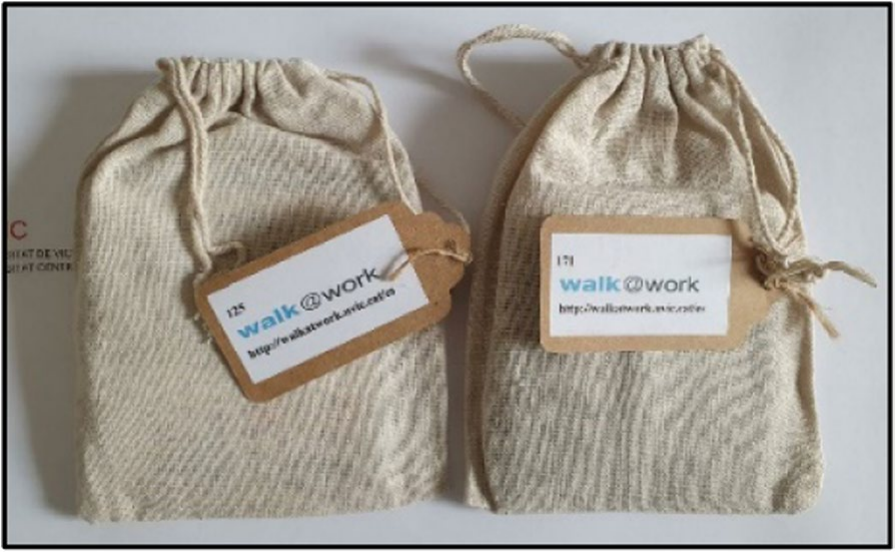
Walk@Work-AppKits provided to nurses of primary health care centres

**Fig. 3:**
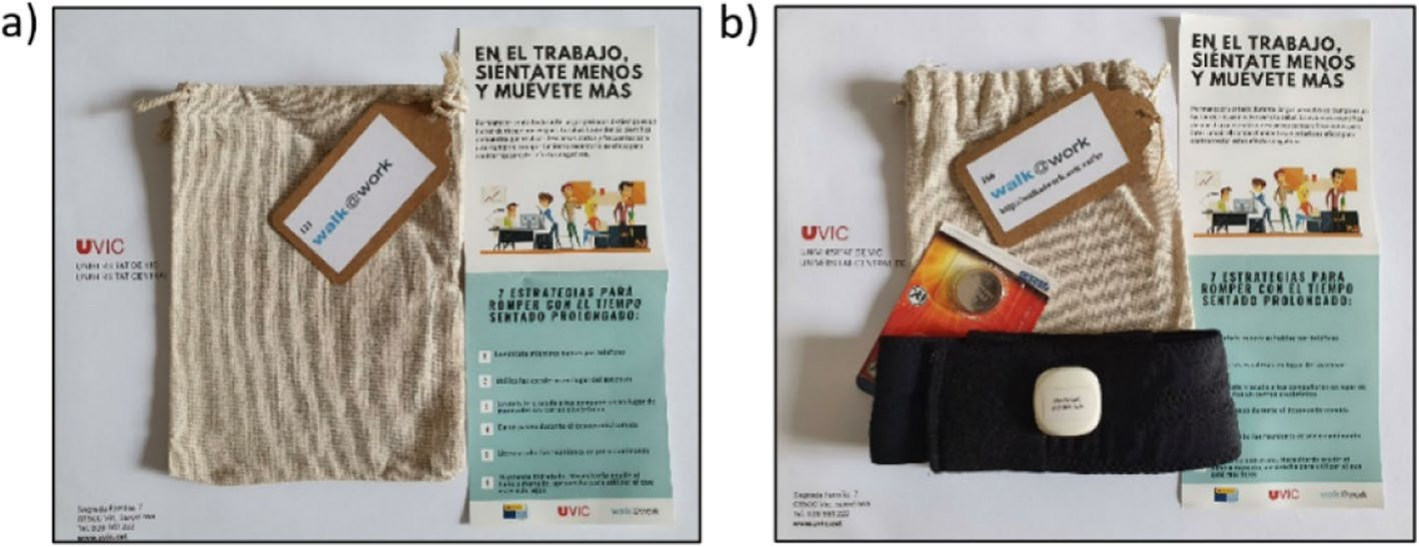
Content of the control (a) and intervention (b) groups kit for Walk@Wor-kApp program

After the number assignment, (i) the research team will blindly provide “Walk@Work-App Kits” for each patient corresponding to either the control or intervention group to nurses. Both kits will have the same look (Fig. 2) but with different content depending on whether the patient belongs to the intervention or control group (Fig. 3). The physician, in charge of recording and monitoring clinical data, will be blinded to the patients’ group randomization. Patients will be blinded to the existence of other patients receiving the intervention or being in a control group. The independent researcher who evaluates the participants at the end of the intervention and at the 6- and 12-month follow-ups will be blinded to the participants’ treatment group assignment. In addition, the person who will carry out the data analysis will not participate in the data collection.

### Intervention

**Fig. 4:**
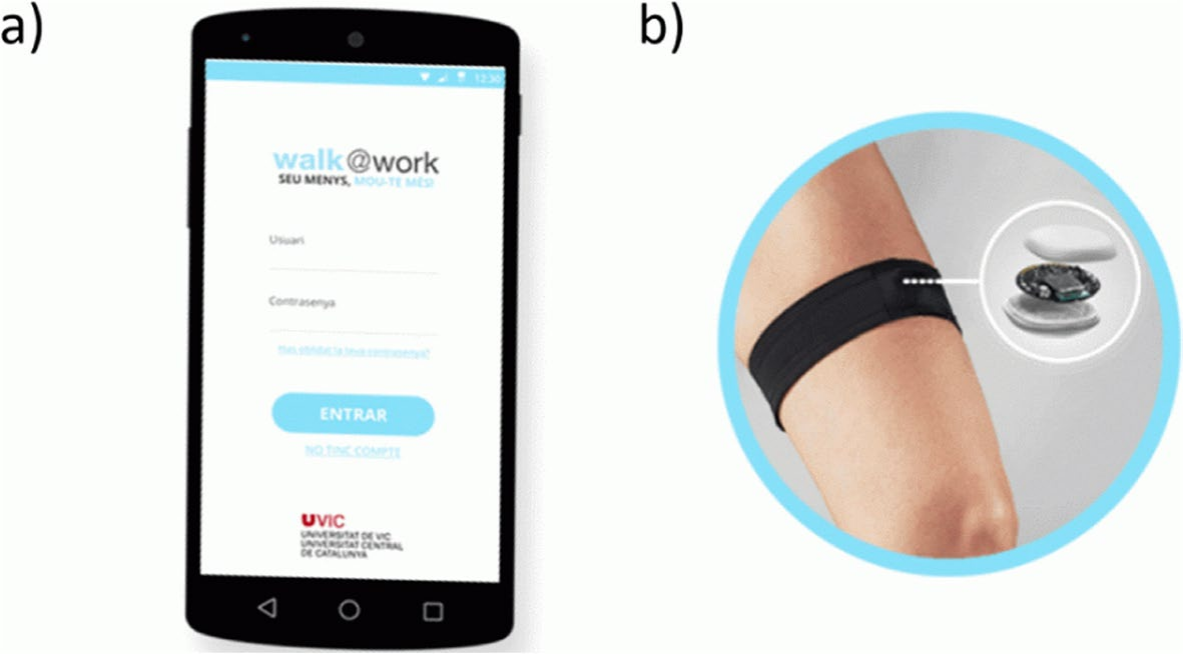
(a) Walk@Work-App interface; (b) the MetaWearC sensor with the case and thigh band

The Walk@Work-Application (W@W-App) is an automated mobile phone and web-based intervention that focuses on decreasing and breaking up prolonged occupational sitting time in desk-based office employees. The W@W-App includes a self-monitoring tool that adds a commercially available sensor (MetaWearC; MbientLab Inc) [62] covered with a waterproof round case and attached via a band to the thigh. The sensor gathers employee’s postural and movement information during working hours. The W@W-App communicates with the MetaWearC external sensor by synchronizing the raw sensor data with the W@W-App software via a low-energy Bluetooth System. Postural and movement data are directly processed and displayed in real time by the app on the phone. Figure 4 depicts the W@W-App (login page) and the MetaWearC sensor.

**Fig. 5:**
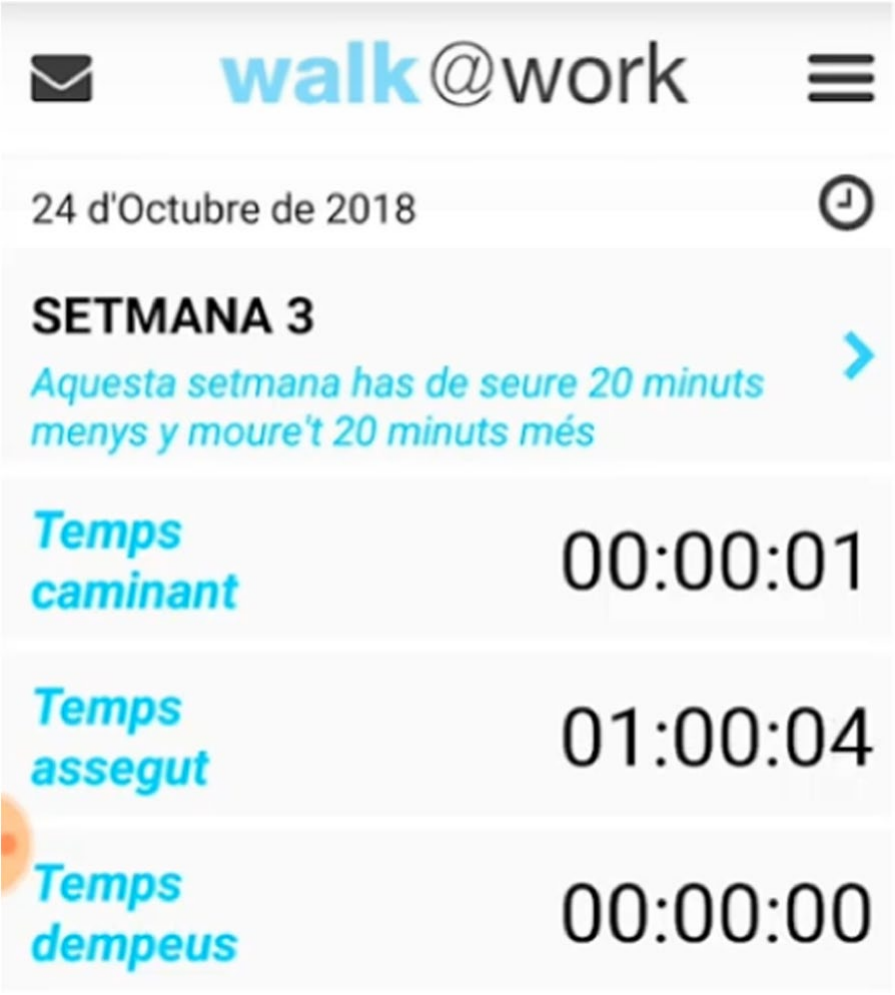
Walk@Work-App interface displaying employees’ occupational stepping, sitting and standing time

The W@W-App+MetaWearC was developed from a previous version [63] to self-monitor and quantify occupational sitting, standing and stepping while offering real-time feedback on these behaviours (Fig. 5). Both are essential components for changing behaviours at the time and place where they occur, as well as for increasing individuals´ awareness and empowerment toward behaviour change [64]. The W@W-App+MetaWearC self-monitoring system has demonstrated a high level of accuracy and validity in determining postural position, providing a low-cost alternative tool for the examination of occupational sitting, standing, stationary and upright times in desk-based office employees [65].

#### Walk@Work-App registration, configuration and instalment

Participants will install and configure the W@W-App, following the guidance provided: (i) registration on the W@W web platform (http://walkatwork.uvic.cat/en/) [66]; (ii) user verification through email; (iii) W@W-App installation and initialization; (iv) recording day and time period configuration (i.e. between 3 and 8 working hours); and (v) recognition of the MetaWearC sensor via Bluetooth. Participants can also read the private policy of the W@W-App on the W@W website. Registration and employee guidance is available on a YouTube tutorial at https://youtu.be/cS0oJ9xPTbo. The W@W-App is available for downloading in Google Play and App Store. After registration and configuration, the complete W@W-App will be installed on the employees´ own mobile phones for 13 weeks, the period during which employees will wear the band with the attached MetaWearC, only during working hours (including the time taken to go to and back from work).

*Description of how the W@W-App programme for changing occupational sedentary time works.*

**Fig. 6:**
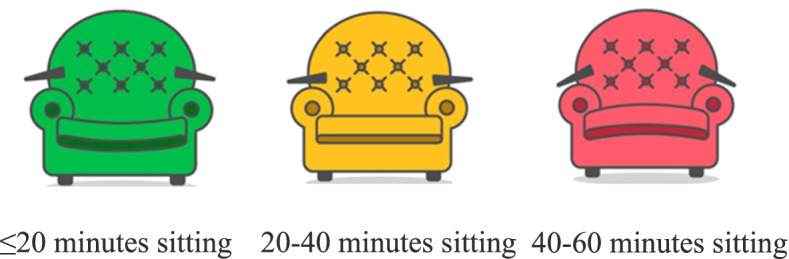
Chair images with feedback displayed on the Walk@Work-App mobile screen

During Week 0, the W@W-App provides self-monitoring features to get baseline measurements for occupational stepping, sitting and standing time and set up a programme baseline with individual targets for each employee. During Weeks 1–12, the app keeps self-monitoring and displaying employees’ occupational activity in real-time, including an emoji of an animated chair at the bottom of the screen. According to the time spent in sitting bouts, the chair changes from a green chair (< 20 min) to a yellow chair (20–40 min) and then to a red chair (40–60 min) (Fig. 6). When sitting time is prolonged for more than one hour, a vibration feature of the mobile phone is activated.

**Fig. 7:**
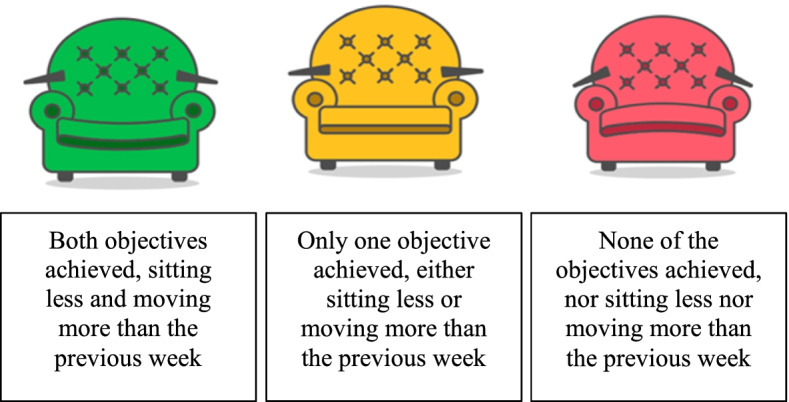
Chair images with feedback on employees’ summary of daily activity displayed on Walk@Work-App

At the end of the working day, the mobile application sends data to the web server and it returns a daily summary message with the support of a chair image reflecting how well the employee has done in reaching the previously set-up individualized goals. In this message, participants can see the chair that represents their daily goals achievement (Fig. 7).

**Fig. 8:**
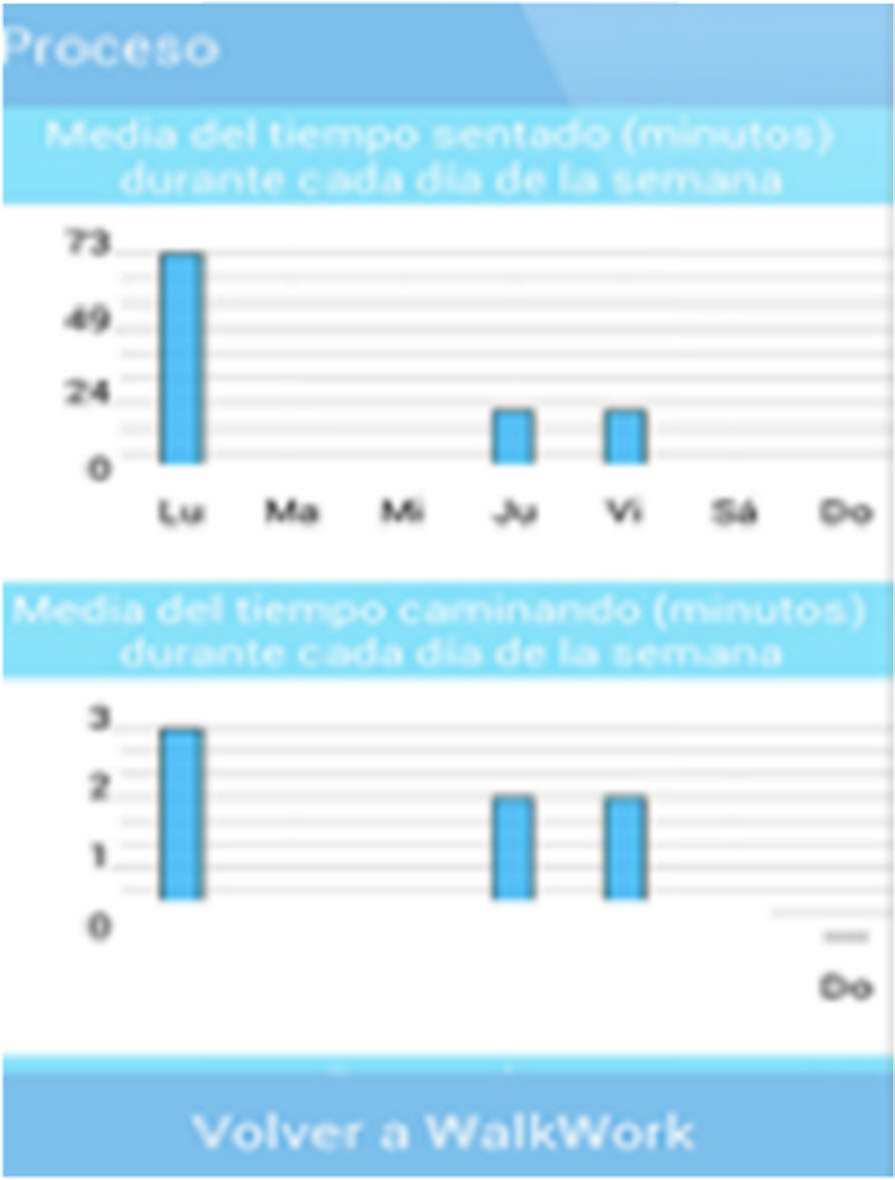
Graphs with employees’ feedback on sitting and moving time displayed on W@W-App mobile screen

**Fig. 9:**
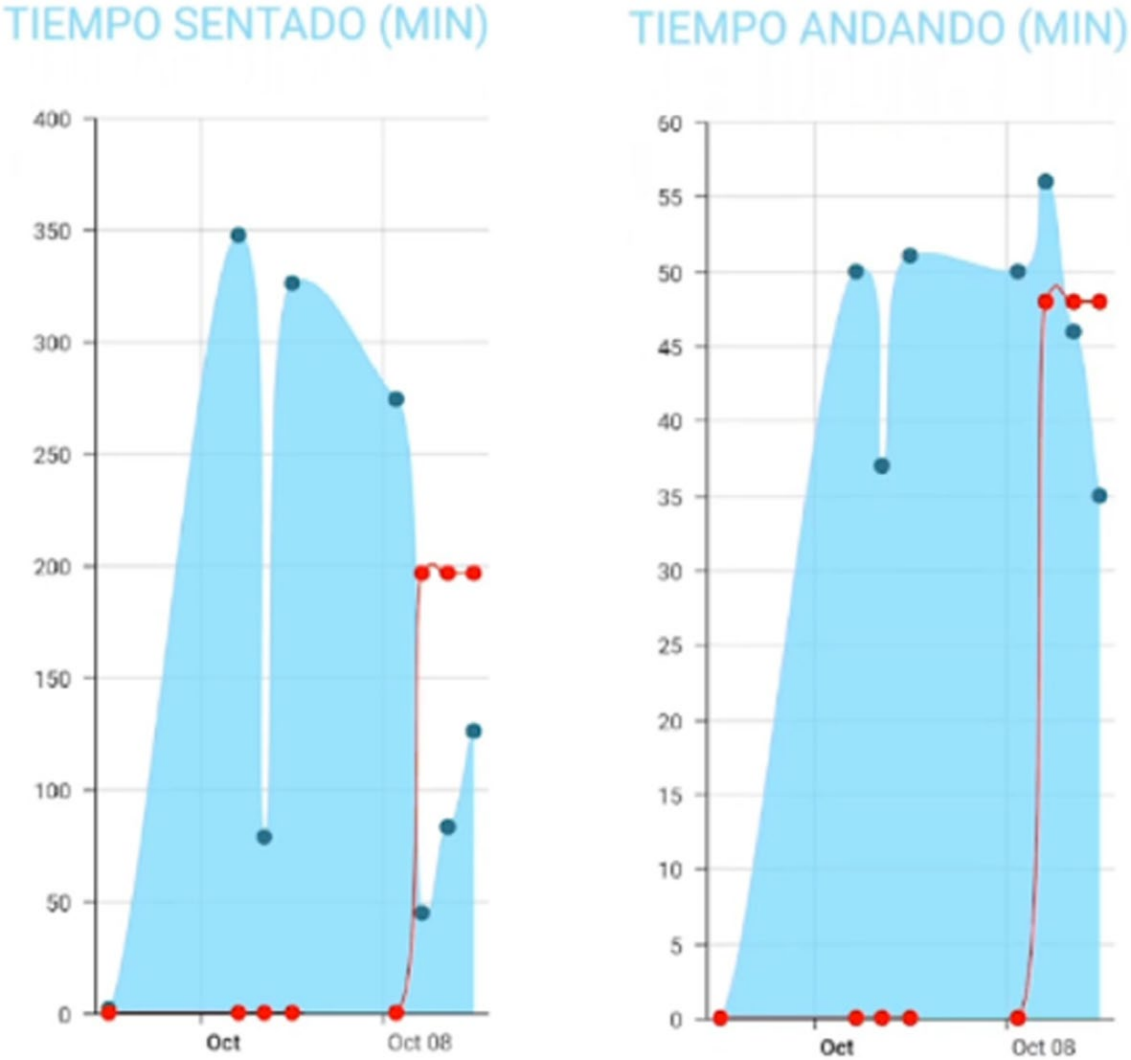
Graphs with employees’ feedback on sitting and moving time displayed on W@W-App website

When one or both targets have been achieved during most days of the week, a congratulations message is sent at the end of the working week with the support of a blue chair image. These weekly motivational messages report the progress made, appearing only when the goals are completed. Additionally, daily graphs provide feedback on employees´ occupational sitting and moving time (Fig. 8). While in the App these graphs can be checked weekly, in the W@W-App website (Fig. 9) the graphs show continuous feedback from week 0 to week 12 in relation to the goals that should be achieved throughout the programme.

Most importantly, during Weeks 1–12 employees have access to automated strategies to sit less and move more at work during an 8-week ramping phase and a 4-week maintenance phase. During this period, fortnight and weekly messages inform employees about the strategies and goals that can be used to change their occupational sedentary behaviour. During the ramping phase, every two weeks employees are challenged to progressively increase their movement by replacing occupational sitting time with 10 minutes (Weeks 1–2), 20 minutes (Weeks 3–4), 30 minutes (Weeks 5–6) or 37 minutes (Weeks 7–8) of moving or stepping above the average baseline (Week 0).

#### Description of the W@W-App strategies

Strategies to achieve the programme goals initially focus on reducing and breaking occupational sitting time by performing work tasks actively, such as active email, breaking up meetings time, getting off the chair briefly to move the joints, walking while talking on the phone and getting up to throw documents in the trash, drink water or print documents (Weeks 1–2). These progress to include short 5-minute walks, for example active parking, active relaxation at work, holding meetings while walking with one or two people or using the farthest bathroom (Weeks 3–4) and then longer walks of 10 minutes by targeting active transport, active breakfasts or including walks in the itinerary to get to work and back home (Weeks 5–6). During weeks 7–8, workers are encouraged to walk with greater intensity by using the stairs, walking briskly or adding three periods of 5–10 minutes of walking per day. Additionally, employees are encouraged to check resources on Twitter that explain in detail the benefits of replacing sitting time for light or moderate physical activity, share with users the barriers they find to reducing sitting time during the work day and show solutions to overcome these barriers. Finally, employees are encouraged to find their own strategies to sit less and move more at work and share them in the W@W-App Twitter. Over these 8 weeks, employees are also encouraged to share their active work experiences on the W@W-App Twitter and share the 5 to 10-minute walks added to their work routine to help others discover various routes and times to walk at work.

During the maintenance phase (weeks 9–12), employees are challenged to keep reducing daily sitting time and increase movement or walking time by 40–60 minutes a day. Strategies to achieve this goal focus on reducing sitting time at weekends, adding commuting or walks in the employees´ immediate environment where they live or work, staying active by helping others and being active or sharing knowledge with others about the benefits experienced from sitting less and being more active. The W@W-App strategies and intervention have been developed from previous versions [67, 68].

#### Description of the W@W-App behaviour change techniques

W@W-App provides a range of effective behaviour change techniques to reduce and break up sitting time and increase physical activity at work [69]. Underpinned by the Behaviour Change Wheel, a theoretically driven framework that incorporates multiple theories of behaviour change [70], the behaviour change techniques included in W@W-App are: (i) Feedback on behaviour and outcomes of behaviour (e.g. providing real-time feedback on the behaviours at the time when they occur); (ii) Self-monitoring of behaviour and outcomes of behaviour (e.g. providing graphics and messages with individual feedback on progress); (iii) Information about health consequences (e.g. providing articles on the health benefits of replacing sitting time with physical activity); (iv) Goal setting (i.e. setting goals every 2 weeks for reducing occupational sitting time and increasing moving); (v) Action Planning (e.g. providing groups of strategies that are progressively planned to increasing the length of the breaks in sitting time); (vi) Social support (i.e. providing a social network for sharing experiences and strategies using twitter or the blog); (vii) Instructions on how to perform the behaviour (e.g. providing detailed instructions of the tasks that can be done to reduce occupational sitting); and (H) Prompts (e.g. using coloured chairs).

### Outcomes

The following outcomes will be assessed on the effectiveness of W@W-App: (i) Objective and subjective habitual and occupational sedentary behaviour, measured by the activPAL3TM device (PAL Technologies Ltd., Glasgow) and the Workforce Sitting Questionnaire (WSQ) [71, 72]; (ii) Objective and subjective habitual and occupational physical activity, measured with the activPAL3TM device and the Spanish Brief Physical Activity Assessment Tool (SBPAAT) [73, 74]; (iii) Glycaemic control variables using glucose, glycosylated haemoglobin A1c (HbA1c); (iv) Anthropometric variables by weight, height, body mass index (BMI), and waist circumference; (v) Lipid profile by total cholesterol, high-density lipoprotein (HDL) and low-density lipoprotein (LDL) and triglycerides; (vi) Systolic and diastolic blood pressure (BP); (vii) Mental well-being measured with the Warwick-Edinburgh Mental Well-being Scale (WEMWS) [75]; and (viii) Work-related outcomes including presenteeism measured with the Work Limitations Questionnaire (WLQ) [76], sickness absence [77], work-related stress (Job Content Questionnaire, JCQ) [78] and impact of work on employees’ health [77].

#### Subjective habitual and occupational sedentary behaviour and physical activity

A seven-day total and domain-specific sitting time questionnaire (Workforce Sitting Questionnaire, WSQ) will assess weekly sitting time (minutes/day) at work *and* while travelling to and from work [71]. These domains will be targeted since Walk@Work-App aims to reduce sitting time (i) at work and (ii) while commuting. This questionnaire has high validity and reliability in the adult population for weekday sitting time at work (r = 0.69–0.74), while it is lower for weekend days across all domains (r = 0.23–0.74) [78]. Forward-backward translation into Catalan and Spanish identified linguistic equivalence [79]. The Spanish version of a Brief Physical Activity Assessment Tool (SBPAAT) will identify participants who are insufficiently active versus those who follow the health recommendations for physical activity [73]. The SBPAAT has showed moderate validity (k = 0.454, 95% CI: 0.402–0.505) and a specificity and sensitivity of 74.3 and 74.6%, respectively. Validity is fair for identifying daily minutes engaged in moderate (r = 0.215, 95% CI: 0.156 to 0.272) and vigorous PA (r = 0.282, 95% CI: 0.165 to 0.391) [73].

### Objective habitual and occupational physical activity

The activPAL3TM will be used to measure and quantify the sedentary behaviour patterns and physical activity of employees across weekdays (working and non-working time) and weekends. Working and non-working times will be established by using participants’ daily records. The activPAL3TM device is a valid measure to quantify body posture and activity patterns during free-living conditions [72]. The device will be attached to participants’ right thigh using a flexible nitrile sleeve and a transparent film (10 × 10 cm of hypoallergenic Tegaderm™ Foam Adhesive Dressing). The waterproof dressing of the activPAL3TM allows participants to wear the monitor continuously for 24 hours per day for 7 complete days. Participants will receive additional dressings and instructions on how to reattach the device if needed. Additionally, participants will be asked to record their daily wake-up time, bedtime, working hours, and any monitor removal time.

Data will be processed using activPAL Professional Software™ (version 7.2.32), Microsoft Excel 2010 (Redmond, WA, USA), and MATLAB v8.4 (MathWorks®, Natick, MA, USA), following previously published protocols [80]. Resulting from the activPAL3TM software output, the following outcomes will be determined: total sitting time, total standing time, total number of sitting bouts and number of sitting bouts with three different lengths (< 20 min, 20–40 min, 40–60 min and > 60 min). Additionally, total time spent in light-intensity physical activity and moderate-to-vigorous physical activity will be determined by using previously validated count-to-activity thresholds [81].

*Positive mental well-being.*

The Warwick-Edinburgh Mental Wellbeing Scale (WEMWBS) will assess positive mental well-being over the previous 2 weeks [75]. The 14-item scale has five response categories: 1 (“None”) to 5 (“All the time”). Responses are added to identify the final score, 14–70, and indicating low to high positive mental well-being. WEMWBS shows high internal reliability (Cronbach’s alpha = 0.93) and 1 week test-retest reliability (r = 0.97) in the Spanish population [82].

#### Presenteeism

The *Work Limitations Questionnaire* (WLQ) will assess performance and the degree to which health problems interfere with the ability to perform job roles [76]. Spanish [83] and Catalan [84] versions of the WLQ have been developed and validated. In the WLQ, respondents self-report levels of difficulty in performing 25 specific job roles across four scales, with scores expressed as an average of responses. The 5-item “Time Scale” addresses difficulty in scheduling demands. For the “Mental-Interpersonal Scale” six items cover difficulty performing cognitive tasks involving the processing of sensory information and interacting with others on the job. The “Output Scale” has five items exploring limitations in meeting demands for quantity, quality and timeliness of completed work. The nine-item “Physical Scale” assesses ability to perform job tasks that involve bodily strength, movement, endurance, coordination and flexibility.

Sub-scales scores are transformed to a 0–100 continuum to represent the amount of time in the previous 2 weeks affected by limited on-the-job performance (from a low to high rate of difficulty). These scales estimate work loss, known as the WLQ index [76], which is the weighted sum of the scores from the WLQ scales. In the present study, the WLQ index will be calculated by adding the scores of three WLQ scales; the “Physical Scale” will be excluded from the current analyses as it was not relevant to these job roles.

#### Job-related mental strain or work-related stress

The Job Content Questionnaire (JCQ) will measure work stressors [78]. This questionnaire follows the demand-control model and includes two dimensions: psychological demands of work and control over work. Work stress is expressed as the result of the interaction between both dimensions. The scale measures a third dimension – social support from co-workers, managers and supervisors – that can act as a moderator of the relationship between the demands of and control over one’s work [78].

The minimum reduced version of the questionnaire includes three dimensions: psychological demands (9 items), control over work (9 items) and support at work (11 items). The possible response categories for each of the items are: totally disagree (1), disagree (2), agree (3) and totally agree (4). The psychological demands dimension assesses workload, the intellectual demands and the time pressure. The control over work dimension assesses the possibility of making decisions, creativity and the application and development of one’s own skills. The support at work dimension assesses the support received from co-workers and supervisors.

The Spanish version of the minimum reduced version shows high reliability and content validity and moderate concept validity for each of the three dimensions [85]. Work stress is expressed as a ratio between psychological demands and control at work, with lower scores indicating less stress. The range of scores for the psychological demands dimensions varies between 12 and 48, while the score for the control at work dimension varies between 24 and 97 [74]. For the social support dimension, co-worker support will be measured by using a 5-item scale, while supervisor support will be measured with a 6-item scale on a 4-point Likert scale (1 = agree; 2 = somewhat agree; 3 = somewhat disagree; and 4 = disagree). Total social support is the sum of co-worker and supervisor support, with higher values reflecting greater perceived social support [78].

#### Employees’ work-related health problems

Six questions from the 6th European Working Conditions Survey will measure work-related health problems [77]. Patients will be asked if they have suffered, in the last 3 months, any of the following health problems: backache, muscle pain in the shoulders, neck and/or upper limbs muscle pain in the lower limbs anxiety, general tiredness or general health problems [77]. The results will be given in percentages.

#### Employees´ sickness absence

Two questions will be asked [77] in relation to the number of work days the patient has missed, in the last 3 months, due to sick leave or health reasons, and how many of these days were caused by work-related health problems (excluding accidents) [77]. The results will be reported as a percentage of patients that have missed a day of work and the average number of days of temporary disability.

### Participant timeline

**Fig. 10:**
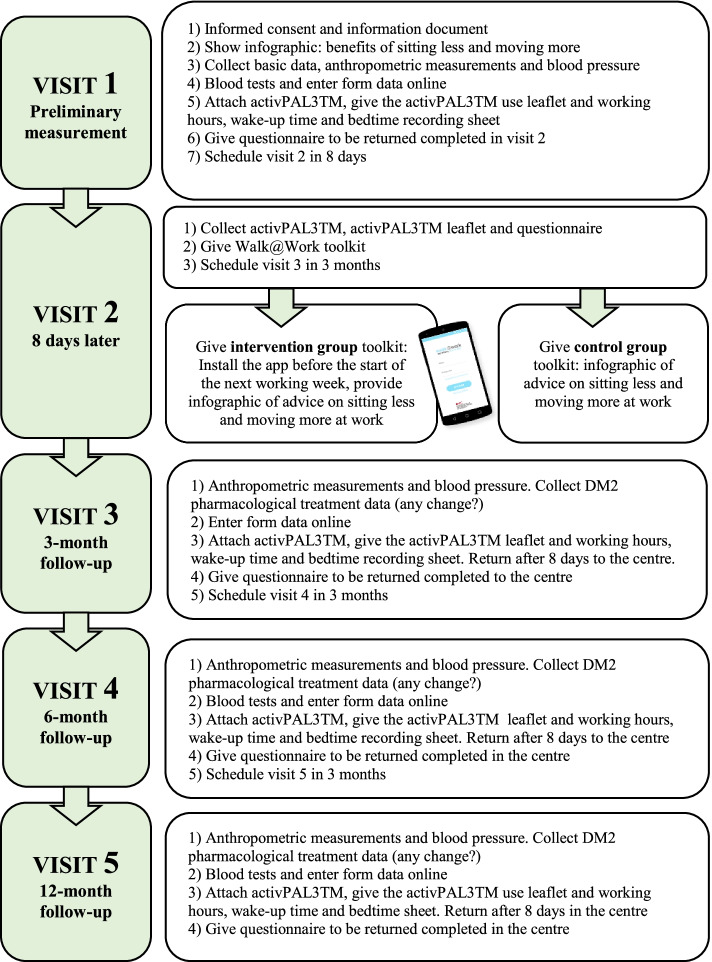
Participants’ timeline for the Walk@Work-App study protocol of a randomized controlled trial

The intervention group will have access to the automated W@W-App for 13 weeks. The control group will be asked to continue their routine daily activities receiving usual health care. All participants will receive the usual health recommendations and general information on the health benefits of sitting less and moving more through an infographic (Additional file 1). Figure 10 shows the planned follow-up visits to be carried out by the healthcare professionals, the variables to be measured and the tasks to be performed in each follow-up visit.

### Data collection methods

The data collection will be carried out at the beginning and at 3, 6 and 12 months using the activPAL3TM device, data from the computerized medical history and a survey. At 6 and 12 months, each patient will have a blood test done for the clinical variables (Table 1). In each measurement, the participants will be summoned in consultation to record the subjective and objective habitual and occupational sedentary behaviour and the subjective and objective habitual and occupational level of physical activity.

**Table 1:** Data collection methods for the Walk@Work-App study protocol of a randomized controlled trial

	Variables	Baseline	Follow-up			Variable type	Objective
		**t = 0** **Months**	**t = 3 Months**	**t = 6 Months**	**t = 12 Months**		
**PARTICIPANT CHARACTERTISTICS**	Socio-labour level	X				QV	Factors that can affect the final result
	Number of working hours	X				CV	
	Changes in glucose-lowering dose within last 3 months		X	X	X	QV	
	Antidiabetic drugs or insulin	X	X	X	X	QV	
	Type of insulin	X	X	X	X	QV	
	Diet	X	X	X	X	QV	
	Change in diet		X	X	X	QV	
**HABITUAL AND OCUPATIONAL SEDENTARY BEHAVIOUR AND PHYSICAL ACTIVITY**	Total sitting time, workday, weekend (ActivPAL3TM)	X	X	X	X	CV	Reduce
	Number of sitting time interruptions workdays, weekend (ActivPAL3TM)	X	X	X	X	CV	Increase
	Sedentary bouts < 20 minutes workdays, weekend (ActivPAL3TM)	X	X	X	X	CV	Reduce long periods of sitting/ increase short periods of sitting
	Sedentary bouts 20–40 minutes workdays, weekend (ActivPAL3TM)	X	X	X	X	CV	
	Sedentary bouts 40–60 minutes workdays, weekend (ActivPAL3TM)	X	X	X	X	CV	
	Sedentary bouts > 60 minutes workdays, weekend (ActivPAL3TM)	X	X	X	X	CV	
	Standing time workdays, weekend (ActivPAL3TM)	X	X	X	X	CV	Reduce
	Activity time workdays, weekend (ActivPAL3TM)	X	X	X	X	CV	Increase
	Light-intensity physical activity workday, weekend (ActivPAL3TM)	X	X	X	X	CV	Increase
	Moderate-to-vigorous physical activity workday, weekend (ActivPAL3TM)	X	X	X	X	CV	Increase
	Sufficiently or insufficiently active (SBPAAT)	X	X	X	X	CV	Increase
	Domain-specific sedentary behaviour (WSQ)	X	X	X	X	CV	Reduce
	Glycaemic index (mg/dl)	X		X	X	CV	Reduce
	HbA1C (%)	X		X	X	CV	Reduce
**LIPID PROFILE**	Total Cholesterol (mg/dl)	X		X	X	CV	Reduce
	HDL (mg/dl)	X		X	X	CV	Increase
	LDL (mg/dl)	X		X	X	CV	Reduce
	Triglycerides (mg/dl)	X		X	X	CV	Reduce
**ANTHROPOMETRIICS**	Weight (Kg)	X	X	X	X	CV	Reduce
	Height (cm)	X	X	X	X	CV	–
	BMI (kg/m^2^)	X	X	X	X	CV	Reduce
	Waist circumferences (cm)	X	X	X	X	CV	Reduce
	*Systolic and Diastolic blood pressure*	X	X	X	X	CV	Reduce
**MENTAL WELL-BEING, WORK OUTCOMES**	Mental well-being (WEMWS)	X	X	X	X	CV	Increase
	Presenteeism (WLQ) and estimated work loss	X	X	X	X	CV	Reduce
	Sickness absence	X	X	X	X	CV	Reduce
	Work control, psychological demands, social support (JCQ)	X	X	X	X	CV	Increase
	Impact of work on employees´ health	X	X	X	X	CV	Improve

The anthropometric variables will be measured with an approved Seca 770 scale and a height measuring rod Seca 222. The BMI will be calculated by dividing the body weight by the square of the height in metres (kg/m^2^). Measurement of the waist circumference, in centimetres, will be completed using a stretch-resistant tape measure and recorded as the midpoint between the lower margin of the lowest palpable rib and the top of the iliac crest. The measurement will be repeated twice. If the measurements are within 1 cm of each other, the average will be calculated; and if the difference exceeds 1 cm, the two measurements will be repeated.

For systolic and diastolic blood pressure (BP), three measurements will be made, using the mean of the last two, with a validated OMRON M3 sphygmomanometer and following the recommendations of the European Society of Hypertension (https://www.eshonline.org/). The degree of metabolic control according to the percentage of HbA1c and basal glucose, lipid profile (total cholesterol total, HDL, LDL, triglycerides) will be measured by a preliminary blood test, and at the 6- and 12-month follow-ups.

At baseline, data from participants’ personal, social and work profile will be collected. Age, sex and social class will be recorded based on the clinical history and data from a questionnaire. A validated scale of occupation as an indicator of social class, according to the British Registrar General classification [86], will be used to record the highest level of completed studies, type of work, length of time working in the company, type of work contract, number of work hours and usual days of work.

Changes in glucose-lowering therapy within the last 3 months, the type of oral antidiabetic drug or insulin, type of insulin, if participants follow a special diet or have modified their diet in the last 3 months will be also recorded. These variables will describe participants and, when the data is processed and if some outlier is observed, to justify possible unusual behaviours. Table 1 shows the variables and its type recorded at each instant: continuous or qualitative variables.

SBPAAT: Brief Physical Activity Assessment Tool; WSQ: Workforce Sitting Questionnaire; BMI: Body mass index; HbA1c: glycosylated haemoglobin A1c; HDL: High-density lipoprotein; LDL: Low-density lipoprotein; WEMWS: Warwick-Edinburgh Mental Well-being Scale; WLQ: Work Limitations Questionnaire; JCQ: Job-related mental strain; Continuous variable (CV), Qualitative variable (QV).

### Statistical methods

A descriptive study of all the measured variables will contain the mean, standard deviation, minimum and maximum value and dispersion coefficient. The homogeneity of the intervention and control group at the initial moment – that is, that there are no statistically significant differences between the two groups in any of the variables measured – will be verified by performing a two-tailed test of means for independent samples for each variable (Additional file 2).

The effect of the mHealth W@W-App programme will be measured by continuous variables (Table 1) which will be used to evaluate the differences between before and after implementing the mHealth programme. In some variables, the programme will be effective if the mean increases in the intervention group while for other variables effectiveness will happen if the mean decreases. For example, the programme will be effective if physical activity increases and total cholesterol decreases. Therefore a one-tailed test of means will be performed. For these continuous variables, the difference of the variable between moment t of the measurement and the initial moment will be calculated, (*X*_*Dt*_ = *X*_*t*_ − *X*_0_), which quantifies whether there have been changes, after t months, associated with the intervention. The intervention has a positive effect if the results of the intervention group are significantly better than those of the control group; that is, if the values of the variable *X*_*Dt*_ are better in the intervention group. The qualitative variables (see Table 1) will be used to check if any patients display unusual behaviour that are due to changes in their medication or diet [86]. Intention-to treat analysis will be used. The statistical tests to be carried out in the event that homogeneity between the groups is not accepted while the procedure is outlined in Additional file 3.

### Sample size

Using the free software G*Power [87], the sample size was calculated based on the power of the test and the effect size. Table 2 shows the results for powers of 0.95 and 0.80 and three effect sizes (i.e. small, medium and large). If the intervention and control group are homogenous, the sample size to be taken for a mean effect is 176 in total; 88 patients to the intervention group and 88 to the control group. With a total sample size of 176, the power of the homogeneity test is 0.91 (Table 2).

**Table 2:** Power function to test homogeneity of the control and intervention group as a function of the effect size

Effect size (∆*μ* = *d* ∙ *σ*))	Power function
Small (*d* = 0.2) (*n* = 1084, power 0.91)	
Medium (*d* = 0.5) (*n* = 176 power 0.91)	
Large (*d* = 0.5) (*n* = 70 power 0.91)	

It is estimated that on average there is a loss of 20% of patients in clinical trials. If the sample size is increased by this percentage, 220 patients will have to be recruited; 110 in the intervention and 110 in the control group. If the losses do not exceed 20%, a test power of 0.95 would be guaranteed for a mean size effect. If the sample size is not increased at baseline, a loss of 20% would mean having a sample size of 140 patients, which would mean a test power of 0.90. The effect size capable of detecting the test performed with a power of 0.95 or 0.8 shows in additional file 4.

### Data monitoring

Operating procedures will be documented in a Standard Operating Procedure manual to standardize the administration of trial conditions, data collection methods, tracking procedures, and checking programming into REDCap (e.g. randomization and administration of tools within specified timeline) [88].

### Ethics and dissemination

This trial will follow the standards of Good Clinical Practice and the principles of the Declaration of Helsinki. The project has been approved by the Clinical Research Ethics Committee of the Primary Care Research Institute Jordi Gol i Gurina with the registration code P18/102. The trial will be reported and patients that volunteer will be asked to sign a written informed consent prior to program participation. Each medical unit participant will be asked for their commitment to the research. The confidentiality of the subjects will be in accordance with regulations of the Organic law of Protection of Personal Data (15/1999 of December 13, LOPD) as well *as the EU General Data Protection Regulation (2016/679, GDPR).* Study results will be reported according to Consolidated Standards of Reporting Trials (CONSORT) recommendations [88] and will also be reported in the ClinicalTrials.gov registry.

## Discussion

Over the last few decades, adults have increased the number of daily hours dedicated to sedentary activities in all areas of life [4]: travel, leisure time, and domestic and work activities. This increase is due to environmental, social, political and cultural factors [23], which result in harmful effects on people’s health, well-being and productivity, and especially people with chronic disease [6]. Given the high prevalence and complexity of chronic diseases associated with sedentary behaviour and physical inactivity such as DM2, it is essential to develop and evaluate new intervention strategies (mHealth) that promote self-care and the sustainability of the health system through the integration of programmes that promote physical activity and the reduction of sedentary behaviour in healthcare. This would contribute to improving the quality of care for people with DM2.

The W@W-App will be applied by healthcare professionals to office workers with DM2, but it could also be applied to other groups of patients with office jobs and chronic diseases associated with sedentary behaviour. The randomized controlled trial of this study will strengthen current evidence on the prescription of mHealth interventions by healthcare professionals to reduce sedentary behaviour and increase habitual and occupational physical activity applied to people with chronic disease. Further, the randomized control trial will examine whether improvement in physical activity and sedentary behaviour leads to both clinical improvement and improvement in well-being and productivity. Therefore, it can provide a practical, cost-effective and accessible intervention that improves adherence to interventions in the lifestyle of people with DM2. The main limitation will be not studying the effect that the intervention could have in the longer term (> 12 months follow-up), which would require a longer study. The main strengths are its evaluation of the impact of a mHealth programme at the clinical level, on mental well-being and on work outcomes and prescribed by healthcare professionals. In addition, it has been designed using behavioural change techniques to reduce sedentary behaviour, and it is the first research of this type to be carried out at the healthcare level, therefore it provides a base for future research in this field.

## Supplementary Information

**Additional file 1.** Infographic with general information on the health benefits of “sitting less and moving more” provided to the control and intervention groups.

**Additional file 2.** Statistical tests to be performed to test for independent samples for each variable.

**Additional file 3.** Statistical tests to be performed in the event that homogeneity between groups is not accepted.

**Additional file 4.** Relationship between sample size and effect size in a mean comparison t test for independent data, one-tailed test. Test power 0.95 and 0.80.

## Abbreviations

DM2: Diabetes Mellitus type 2
WHO: World Health Organization
mHealth: mobile Health
OSPAQ: Occupational Sitting and Physical Activity Questionnaire
W@W-App: Walk@Work-Application
WSQ: Workforce Sitting Questionnaire
SBPAAT: Spanish Brief Physical Activity Assessment Tool
HbA1c: Glycosylated Hemoglobin A1c
BMI: Body Mass Index
HDL: High-Density Lipoprotein
LDL: Low-Density Lipoprotein
BP: Blood Pressure
WEMWS: Warwick-Edinburgh Mental Well-being Scale
WLQ: Work Limitations Questionnaire
JCQ: Job Content Questionnaire
CV: Continuous Variables
QV: Qualitative Variable
CONSORT: Consolidated Standards of Reporting Trials
