# Supplementary material for: Effectiveness of a healthcare-based mobile intervention on sedentary patterns, physical activity, mental well-being and clinical and productivity outcomes in office employees with type 2 diabetes: study protocol for a randomized controlled trial
Source: BMC Public Health. 2022 Jun 29;22:1269. doi: 10.1186/s12889-022-13676-x (PMC9244393; doi:10.1186/s12889-022-13676-x)

Additional file 1: Infographic with general information on the health benefits of “sitting less and moving more” provided to the control and intervention groups.


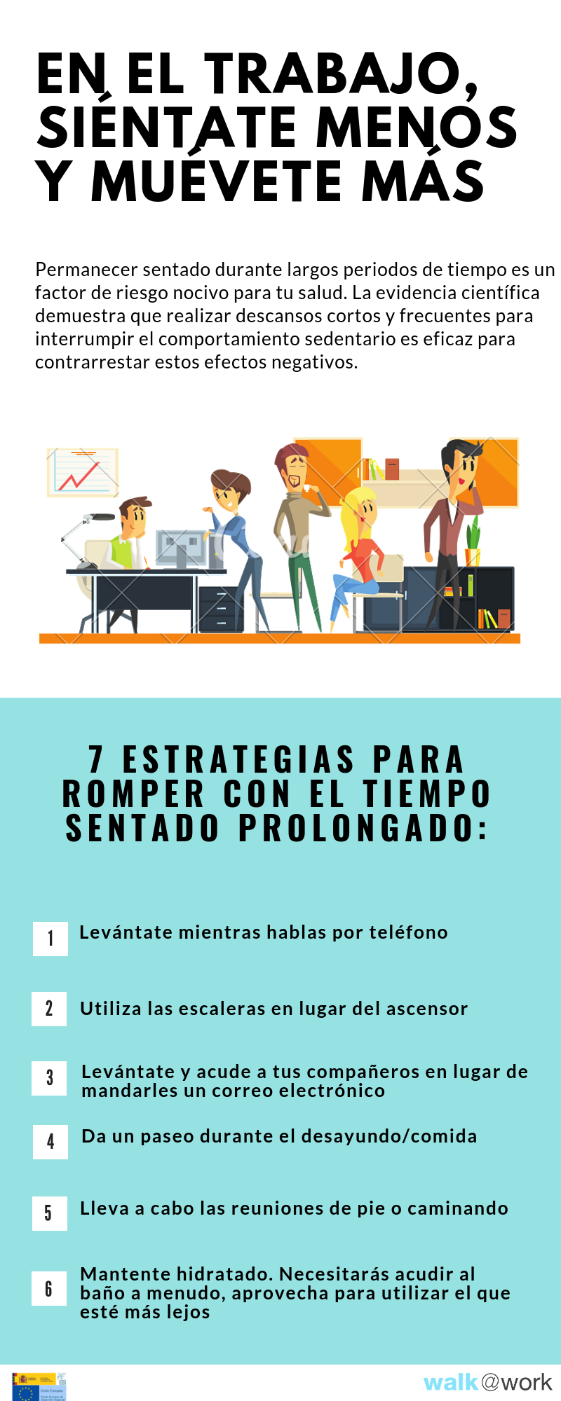

Supplement: Supplementary file 1 — Additional file 1. Infographic with general information on the health benefits of “sitting less and moving more” provided to the control and intervention groups. [file 12889_2022_13676_MOESM1_ESM.docx]
