# Supplementary material for: Effectiveness of a healthcare-based mobile intervention on sedentary patterns, physical activity, mental well-being and clinical and productivity outcomes in office employees with type 2 diabetes: study protocol for a randomized controlled trial
Source: BMC Public Health. 2022 Jun 29;22:1269. doi: 10.1186/s12889-022-13676-x (PMC9244393; doi:10.1186/s12889-022-13676-x)
Supplement: Supplementary file 2 — Additional file 2. Statistical tests to be performed to test for independent samples for each variable. [file 12889_2022_13676_MOESM2_ESM.docx]

Additional file 2**:** Statistical tests to be performed to test for independent samples for each variable.

| **Type of variable** | **Objective of the test** | **Null hypothesis** | **Statistical treatment** |
| --- | --- | --- | --- |
| Continuous variable | Check that there is no difference between IG and CG | Mean_GI_ = Mean_GC_ | Two-tailed t test for independent data. |
| The homogeneity of IG and CG is accepted | | | |
| Continuous variable | Check if the mean value in the IG has decreased or increased | Mean_GI_ > Mean_GC_  Mean_GI_ < Mean_GC_ | One-tailed t test for independent data. |
| The homogeneity of IG and CG is not accepted; see procedure in Additional file 3 | | | |
