# Supplementary material for: Effectiveness of a healthcare-based mobile intervention on sedentary patterns, physical activity, mental well-being and clinical and productivity outcomes in office employees with type 2 diabetes: study protocol for a randomized controlled trial
Source: BMC Public Health. 2022 Jun 29;22:1269. doi: 10.1186/s12889-022-13676-x (PMC9244393; doi:10.1186/s12889-022-13676-x)
Supplement: Supplementary file 3 — Additional file 3. Statistical tests to be performed in the event that homogeneity between groups is not accepted. [file 12889_2022_13676_MOESM3_ESM.docx]

Additional file 3**:** Statistical tests to be performed in the event that homogeneity between groups is not accepted.

If homogeneity cannot be accepted, all participants will become part of the IG, and the data obtained will be treated statistically as paired data. The individual results will be compared to see if they have improved with the intervention; that is, if the value at moment $X_{t}$ is better than at the initial moment, $X_{0}$ (see Table additional file 3).

Table additional file 3. Statistical tests to be carried out for the continuous variables measured

| Objective | Test | Statistical test to be carried out |
| --- | --- | --- |
| Check if the mean value has decreased or increased since the initial values. | Mean_before_ > Mean_after_  Mean_before_ < Mean_after_ | One-tailed t test for related data. |

The sample sizes to be taken to carry out the test of Table A1 based on the effect size to be detected and the power of the test is shown in Table A2. These sizes are lower than those required if homogeneity of the intervention and control groups is accepted, which is 176, thus ensuring a sample size that is sufficient to detect differences between groups.

Table A2. Sample size based on effect size and power of the test.

| Effect size  ($\Delta\mu=d\cdot\sigma)$ | Sample size, $n.$ | | Power function ($\alpha=0.05).$ |
| --- | --- | --- | --- |
|  | Power of test  0.95 | Power of test  0.80 |  |
| Small  $(d=0.2)$ | 272 | 156 | 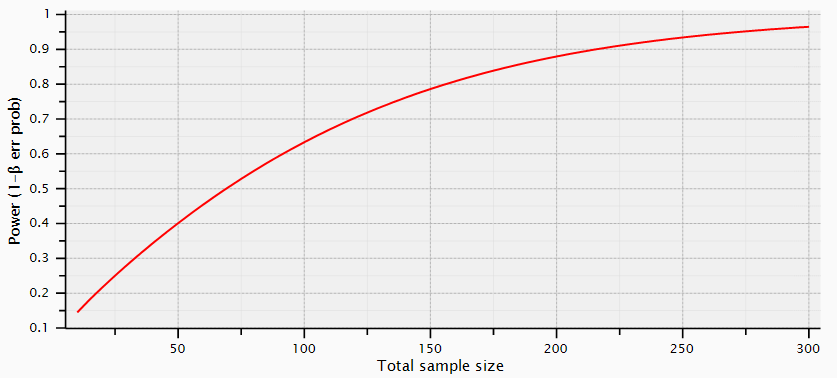 |
| Medium  $(d=0.5)$ | 45 | 27 | 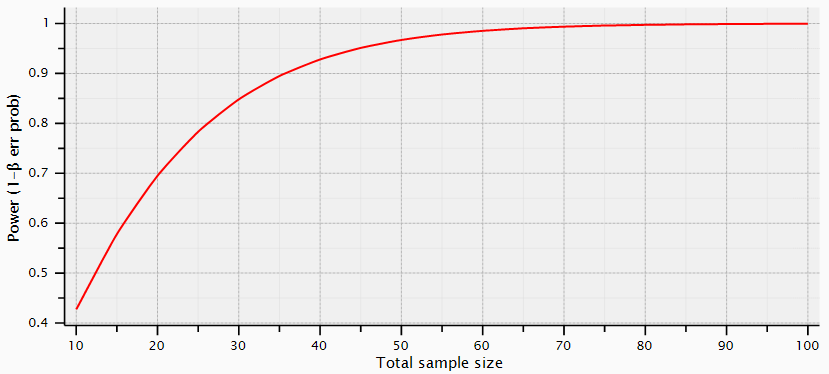 |
| Large  $(d=0.8)$ | 19 | 12 | 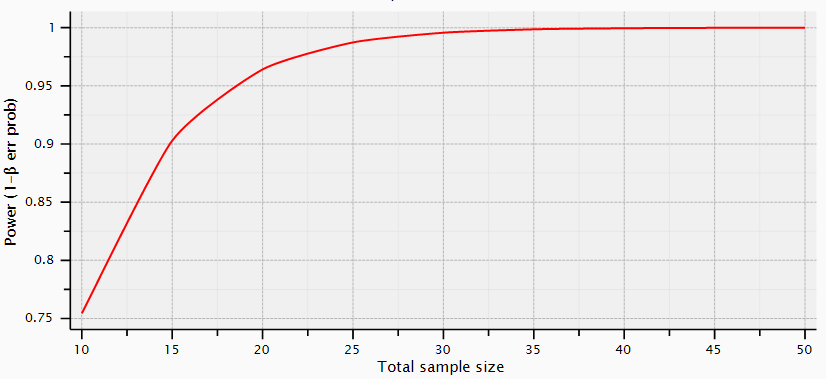 |

.
