# Supplementary material for: Effectiveness of a healthcare-based mobile intervention on sedentary patterns, physical activity, mental well-being and clinical and productivity outcomes in office employees with type 2 diabetes: study protocol for a randomized controlled trial
Source: BMC Public Health. 2022 Jun 29;22:1269. doi: 10.1186/s12889-022-13676-x (PMC9244393; doi:10.1186/s12889-022-13676-x)
Supplement: Supplementary file 4 — Additional file 4. Relationship between sample size and effect size in a mean comparison t test for independent data, one-tailed test. Test power 0.95 and 0.80. [file 12889_2022_13676_MOESM4_ESM.docx]

Additional file 4: Relationship between sample size and effect size in a mean comparison t test for independent data, one-tailed test. Test power 0.95 and 0.80.

**
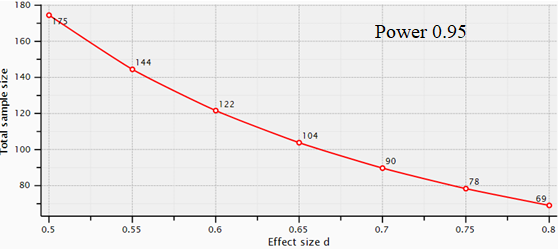

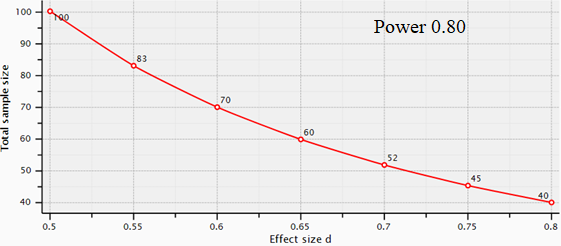
**

.
